# Dynamic profiling of immune microenvironment during anti-PD-1 immunotherapy for head and neck squamous cell carcinoma: the IPRICE study

**DOI:** 10.1186/s12885-023-11672-x

**Published:** 2023-12-08

**Authors:** Carinato Hélène, Ombline Conrad, Carole Pflumio, Christian Borel, Manon Voegelin, Alexandre Bernard, Philippe Schultz, Mihaela-Alina Onea, Alain Jung, Sophie Martin, Mickaël Burgy

**Affiliations:** 1 Department of Medical Oncology, Institut de Cancérologie Strasbourg Europe France, Strasbourg, France; 2 Laboratory of Bioimaging and Pathology, University of Strasbourg, UMR7021 CNRS, Strasbourg, France; 3 Department of Clinical Research, Institut de Cancérologie Strasbourg Europe France, Strasbourg, France; 4 Department of Otolaryngology and Cervico-Facial Surgery, Strasbourg University Hospital France, Strasbourg, France; 5 Department of Pathology, Strasbourg University Hospital France, Strasbourg, France; 6 Laboratory of Tumor Biology, Institut de Cancérologie Strasbourg Europe, Strasbourg, 67200 France

**Keywords:** Head and neck squamous cell carcinoma, Spatial transcriptomic, Immune response, HNSCC tumour microenvironment, Response biomarker

## Abstract

**Background:**

Immune checkpoint inhibitors of programmed cell death protein 1 (PD-1) represent a significant breakthrough in treating head and neck squamous cell carcinoma (HNSCC), with long-lasting responses and prolonged survival observed in first- and second-line therapy. However, this is observed in < 20% of patients and high primary/secondary resistance may occur. The primary objective of the identification of predictive factors for the response to anti-PD-1 immunotherapy in head and neck squamous cell carcinoma (IPRICE) study is to identify predictive factors of response to anti-PD-1 immunotherapy.

**Methods:**

The IPRICE study is a single-center, prospective, non-randomized, open-label, and interventional clinical trial. Liquid and tumor biopsies will be performed in 54 patients with recurrent/metastatic (R/M) HNSCC undergoing anti-PD-1 immunotherapy alone to compare the evolution of gene expression and immunological profile between responders and non-responders. We will use a multidisciplinary approach including spatial transcriptomics, single seq-RNA analysis, clinical data, and medical images. Genes, pathways, and transcription factors potentially involved in the immune response will also be analyzed, including genes involved in the interferon-gamma (IFN-γ) pathway, immunogenic cell death and mitophagy, hypoxia, circulating miRNA-mediated immunomodulation, cytokines, and immune repertoire within the tumor microenvironment (TME). With a follow-up period of 3-years, these data will help generate effective biomarkers to define optimal therapeutic strategy and new immunomodulatory agents based on a better understanding of primary/secondary resistance mechanisms. Tumor biopsy will be performed initially before the start of immunotherapy at the first tumor assessment and is only proposed at tumor progression. Clinical data will be collected using a dedicated Case Report Form (CRF).

**Discussion:**

Identifying predictive factors of the response to anti-PD-1 immunotherapy and optimizing long-term immune response require a thorough understanding of the intrinsic and acquired resistance to immunotherapy. To achieve this, dynamic profiling of TME during anti-PD-1 immunotherapy based on analysis of tumor biopsy samples is critical. This will be accomplished through the anatomical localization of HNSCC, which will allow for the analysis of multiple biopsies during treatment and the emergence of breakthrough technologies including single-cell RNA sequencing (scRNA-seq) and spatial transcriptomics.

**Trial registration:**

Clinicaltrial.gov. Registered April 14, 2022, https://www.clinicaltrials.gov/study/NCT05328024.

## Background

Head and neck squamous cell carcinoma (HNSCC) arising on the mucosal surfaces of the oral cavity, sinonasal cavity, pharynx, and larynx represents the eighth most common cancer worldwide, with an estimated annual incidence and mortality of around 880,000 and 445,000 cases, respectively [1]. Tobacco, excessive alcohol consumption, and human papillomavirus (HPV) infection are the main risk factors of HNSCC. The prognosis for HPV-associated oropharyngeal cancer is more favorable due to a better response to chemotherapy and radiotherapy, and fewer comorbidities [2, 3], whereas the prognosis for recurrent or metastatic (R/M) HNSCC is poor. In 2008, the addition of cetuximab to conventional platinum/5-fluorouracil (5-FU) chemotherapy (EXTREME regimen) improved overall survival as seen in long-term responders, although it was a marginal improvement (48 months progression-free survival (PFS) rate < 3%) [4, 5]. Immune checkpoint inhibitors of programmed cell death protein 1 (PD-1) are an important breakthrough with prolonged duration of response and survival observed in both first- and second-line therapies (5 year-overall survival (OS) rate 15.4% for pembrolizumab monotherapy for the combined positive score (CPS) ≥ 1 arm from the KEYNOTE 048 cohort, Makoto Tahara et al. ESMO 2022) [6, 7]; but, this duration of response is observed in < 20% of the patients. The only predictive marker currently available is the expression of PD-L1, according to the CPS, which has been shown to correlate with survival [7]. However, strong primary/secondary resistance is observed, and tumor response is unpredictable (with an objective response rates (ORR) of 23.3% for pembrolizumab monotherapy in the CPS ≥ 20 arm). Consequently, patients with severe symptoms awaiting an urgent therapeutic response require treatments including chemotherapy to reduce life-threatening progression at the cost of increased toxicity [7]. In addition, a growing number of studies are reporting a long-term survival benefit from cetuximab-based chemotherapy after failure of immunotherapy [8–10]. Therefore, immune checkpoint inhibitors appear to be the only current treatment providing effective clinical responses. These observations underline the main challenge in cancer immunotherapy: optimizing long-term survival requires personalized approaches based on composite biomarkers to define the optimal therapeutic strategy and discover new drugs that can generate immunity in patients who do not have a strong immune response [11]. The development of biomarkers and new therapeutic agents to overcome primary/secondary resistance requires an optimum knowledge of the complex interactions between cancer and the immune system involving molecular and cellular drivers of immune escape. This is considered to be a major challenge facing cancer immunotherapy [11]. However, this objective is complicated by the difficulty of studying not just immune cells but rather an “orchestra of immune and non-immune players” evolving during systemic cancer therapy (Girolami et al. 2023 Journal of Personalized Medicine). Currently, the classification of tumor immunity is almost exclusively based on the spatial distribution of CD8 + T cells in the tumor microenvironment (TME), which are targeted by immune checkpoint inhibitors of PD-1 thereby enhancing their anti-tumor functions [12]. A gradient of three immunophenotypes associated molecular pathways is observed: inflamed tumors including tumor infiltrating lymphocytes (TILs) and B cells in the TME in close proximity to tumor cells; immune-excluded tumors including immune cells distant from tumor cells in the tumor stroma; and immune desert tumors devoid of T cell infiltration. Using gene expression of HNSCC tumor data obtained from The Cancer Genome Atlas (TCGA) database, another immune-infiltrating signature-based classification was recently proposed with three immunophenotypes including other immune cells: cold, lymphocyte (enrichment for CD4 + T cells, CD8 + T cells, and B cells) similar to the previous classification, and myeloid dendritic (DC) signatures (enrichment of neutrophils, macrophages, monocytes, DCs, and T regulatory (Treg) cells) [13]. Thus, classifying TME based on immune cell infiltration alone excludes major actors in cancer immunity such as stromal cells including carcinoma associated fibroblasts (CAF) or tertiary lymphoid structures (TLS) representing promising targets for cancer treatment [14]. Notwithstanding these limitations, classification based on immune cells infiltration provides a reference framework for research describing specific molecular mechanisms for each immunophenotype such as interferon-gamma (IFN-γ) signaling associated with inflamed tumors and transforming growth factor-β (TGF-β) signaling with immune-excluded tumors. Immunotherapy resistance observed in cold tumor may be related to impaired antigen processing (Human leukocyte antigens (HLA) and/or β2 microglobulin loss or downregulation, and elimination of neoantigens through copy-number loss), loss of T-cell priming mainly because of the inhibition of T cells generation by DCs in desert tumor, or the absence of preexisting antitumor T cells infiltration (T-cell-excluded tumors) [15]. Therefore, to enhance antitumor immune responses, next generation immunotherapy should promote lymphocyte activation by tumor antigens and antigen-presenting cells (APC), differentiation and infiltration of lymphocytes in the TME, and tumor cell recognition. However, new therapeutic drugs still have limitations in achieving long-term responses and survival in the majority of patients. This is illustrated in HNSCC by the absence of new therapeutic agents since the arrival of PD-1/PDL-1 blockade drugs and the large number of failures in clinical trials testing combination treatment with immune checkpoint inhibitors in recent years: INDUCE-3 and INDUCE-4 trials investigating feladilimab (inducible T cell co-stimulatory agonist) in combination with pembrolizumab (press release GSK April 14, 2021), INTERLINK-1 study evaluating monalizumab (inhibitor targeting natural killer cells group 2A) in combination with cetuximab (press release Innate pharma Jan 8, 2022), CP-MGA271-06 study with enoblituzumab (anti-B7-H3 mediating antibody-dependent cellular cytotoxicity (press release MacroGenics Jul 8, 2022), Active8 trial with motolimod (TLR8 agonist), and ATHENA trial evaluating the combination of atezolizumab and bevacizumab [16, 17]. Although there are promising studies with drugs targeting immune checkpoint receptors (TACTI-002 Part C: A phase II study of eftilagimod alpha, a soluble lymphocyte-activation gene 3 (LAG-3) protein and pembrolizumab), or immunomodulators (BCA101, bifunctional epidermal growth factor receptor (EGFR)/TGFβ inhibitor and pembrolizumab), these are preliminary studies with a small sample size and a tumor response of less than one in two patients [18, 19].

The immune microenvironment is a dynamic structure in a complex interaction with an evolving cancer, but is affected by specific oncology treatments, particularly the immune checkpoint inhibitors. Therefore, a differential evolution of the TME between responders and non-responders induced by cytotoxic T-lymphocyte–associated antigen 4 (CTLA-4) and PD-1 blockade have been observed in a longitudinal study of metastatic melanoma patients [20]. Significantly, differences in TME composition such as number of CD4 + or CD8 + T cells are increased during on-treatment than before suggesting that predictive biomarkers based on immune signatures should be evaluated in on-treatment tumor samples. Few other recent studies have confirmed temporal changes of the TME during PD-1 inhibitor therapy between immune subpopulations but also for tumor mutational burden, T-cell receptor (TCR) repertoire, immune-related genes, proliferation-associated and chemokine genes, neoantigen immunogenicity landscapes, and tumor clonal populations [21–25]. However, how immune checkpoint inhibitors shape the TME and how they interact with cancer cells remain poorly understood. Therefore, the majority of studies only provide a snapshot of the immune landscape because dynamic profiling of the TME requires biological samples not often available in clinical practice. Profiling of the TME during anti-PD-1 immunotherapy may be essential to accelerate the understanding of the mechanisms that underlie primary and secondary resistance [26–28]. Besides existing immunotherapy, studies also suggest the importance of a deeper understanding of dynamic neoantigen profiles for vaccine development [26]. Targeting antigen selection to ensure high immunogenicity and tumor-specificity is a complex process considering mutation calling, clonality, HLA typing and binding affinity, antigen processing, and similarity to self [29]. Genomic and transcriptomic data may facilitate this selection step by prioritizing the antigens maintained despite tumor evolution and neoantigens silenced in immune-excluded TME [26]. This latter mechanism could explain tumor responses observed on salvage chemotherapy after progression with immune checkpoint inhibitors used in first-line treatment, but our proposal in this study is yet to be investigated [30]. Collecting consecutive clinical tumor biopsies remains a challenge when the treatment is ongoing, suggesting that basic research in cancer immunology and the development of cancer immunotherapy agents are largely based on treatment-naive tumors. However, most patients with incurable HNSCC eligible for immune checkpoint inhibitors had locoregional recurrence occurring in a previously irradiated area, which probably induce a change in TME. However, in the case of HNSCC, tumors are often visible and palpable, and biopsies can therefore be easily processed, allowing a longitudinal TME profiling.

A differential TME-related gene expression analysis between anti-PD-1/PD-L1 immunotherapy responders and patients with a progressive disease requires the use of breakthrough technologies. Indeed, deep analysis of the main cellular subsets including tumor cells, tumor-associated stromal cells, and tumor-infiltrating immune cells should consider their position, their organization in the TME, their state, function and cellular phenotype, their interaction with neighboring cells, and the changes over time caused by immunotherapy. Among these new technologies, single-cell RNA sequencing (scRNA-seq) offers the highest cellular resolution but requires tissue dissociation resulting in the loss of spatial information. Spatial transcriptomics (ST), named “Method of the Year 2020” by Nature Methods, is another powerful technique. It preserves the spatial context of biological data and allow the visualization of gene expression distributions. It is suitable for small samples but is limited by low cellular resolution [31, 32]. Thus, scRNA-seq and ST are two complementary technologies that will be performed in this study. Differential gene expression analysis between responders and non-responders to anti-PD1/PD-L1 immunotherapy will focus on immune-related genes such as immune checkpoint genes, but also on genes that play important roles in TME including hypoxia-related genes, ΔNp63 (potentially implicated in the recruitment of myeloid-derived suppressor cells and overexpressed in HNSCC), immunogenic cell death genes, and mitophagy-related genes, which may be targeted to improve the effectiveness of cancer immunotherapy [33–35]. A particular focus will be made on IFN-γ mediated signaling that may result in both protumoral (immunosuppression, angiogenesis, and tumor cell proliferation) and antitumoral (tumor cell killing, T cell activation, immune cell proliferation, and promotion of antigen presentation) activities indicating the emphasis on its therapeutic modulation [36]. These conflicting activities may be related to the duration and the importance of IFN-γ signaling which are affected by tumor burden, immune cell infiltration characteristics, and cancer treatment (immunotherapies and chemotherapies) [36]. Although producers of IFN-γ and the different effects of IFN-γ in the TME are well documented, lots of questions remain on its involvement in the primary/secondary resistance and on its complete and long-lasting response to immune checkpoint inhibitors. Understanding these mechanisms also requires dynamic analysis of IFN-γ production in the TME undergoing immunotherapy.

Thus, analysis of biopsy samples of patients undergoing immunotherapy might contribute to a better understanding of resistance process but with inherent limitations. These include the invasive nature of the analysis with uncertainties related to patients compliance issues and importantly, the intra-tumor heterogeneity with disparate levels of immune infiltration leading to underestimation of the tumor genomics landscape. Conversely, liquid biopsy may overcome tumor heterogeneity in addition to being noninvasive, allowing continuous monitoring of circulating tumor DNA (ctDNA), circulating tumor cells (CTCs), exosomes, miRNAs, circulating immune cell, and TCR repertoire [37]. Studies have already documented the prognostic ability of ctDNA to detect HNSCC recurrence and/or metastasis [38]. However, there are no prevalent hotspot mutations in HNSCC, only a wide spectrum of mutations including for TP53 tumor suppressor gene which is the most frequent of all somatic genomic alterations in HNSCC [39]. Thus, ctDNA monitoring requires personalized trackable mutations.

The same would apply to miRNAs, a class of endogenous non-coding RNAs involved in post-transcriptional regulation of gene expression and playing an important role in chemo-resistance of cancer cells [40]. Cancer-associated circulating miRNAs are secreted both by immune and tumor cells, suggesting potential predictive value and therapeutic interest [41, 42]. Lastly, understanding the role of circulating cytokines in cancer immunotherapy was a major development that led to the approval of recombinant IFN-α and interleukin (IL)-2 for the treatment of several malignant diseases [43]. Current challenges include suppression of immunosuppressive cytokines, limitation of systemic proinflammatory effects, and to a lesser extent, biomarker development [43]. Thus, tumoral immunity is characterized by a complex interaction between the TME and the peripheral immune system that requires a deeper understanding. Regarding liquid biopsy, the proposed study will focus on the exploration of circulating miRNA-mediated immunomodulation and circulating cytokines.

In this single-center prospective study, we propose to combine liquid biopsy, tissue biopsy, and clinical data at key time points during the management of patients undergoing anti-PD-1 immunotherapy for R/M HNSCC. We will compare the evolution of gene expression profile between responders and non-responders, using spatial transcriptomics analysis among other technologies.

## Methods/design

### Study design

**Fig. 1:**
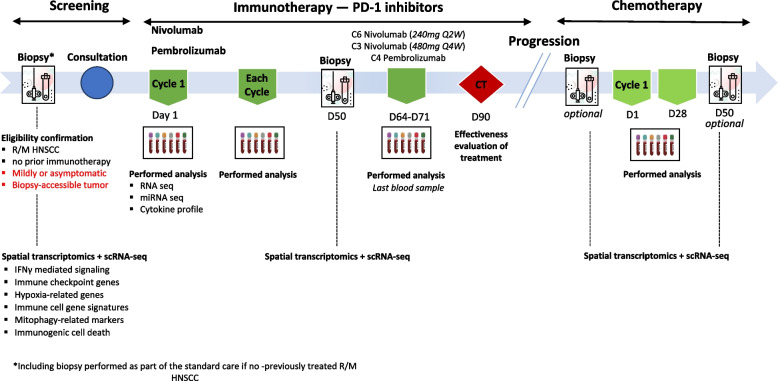
IPRICE study design

The IPRICE study is a single-center, prospective, non-randomized, open-label, and interventional clinical trial of patients managed at the Cancer Institute of Strasbourg (ICANS) Europe (Strasbourg). Study inclusion period will be 3 years; each patient will be followed up for 3 years and the total study duration will be 6 years. The study design is depicted in Fig. 1. The IPRICE study and this manuscript have been written in accordance with standard protocol items, namely recommendations for interventional trials (SPIRIT).

### Study objectives and endpoints

The main objective of this study is to identify effective biomarkers of response to PD-1 checkpoint blockade using combinatorial omics analysis including spatial transcriptomics, scRNA-seq, and miRNomics. The primary endpoint is the prospective validation of the IFN-γ–related gene expression signature to predict response to PD-1 checkpoint blockade (overall response including complete response (CR) and partial response (PR)). Tumor response was defined according to the Response Evaluation Criteria In Solid Tumor (RECIST) criteria. Secondary outcomes are the prospective validation of potential response biomarkers (immunogenic cell death and mitophagy-related markers, expression of hypoxia-related markers, and circulating miRNA-mediated immunomodulation). Exploratory outcome measures will include dynamic and differential profiling of the immune repertoire in the TME between responders and non-responders.

### Study population

Eligibility criteria are described in Table 1. The IPRICE study will focus on adult patients diagnosed with R/M HNSCC considered incurable by local therapies and those eligible for treatment with pembrolizumab alone or nivolumab according to the European Marketing Authorization and managed at the Cancer Institute of Strasbourg (ICANS) Europe (Strasbourg). The patients will be enrolled in the study after screening based on the below mentioned criteria. An identification number will be assigned to each patient to be used throughout the study.

**Table 1:**
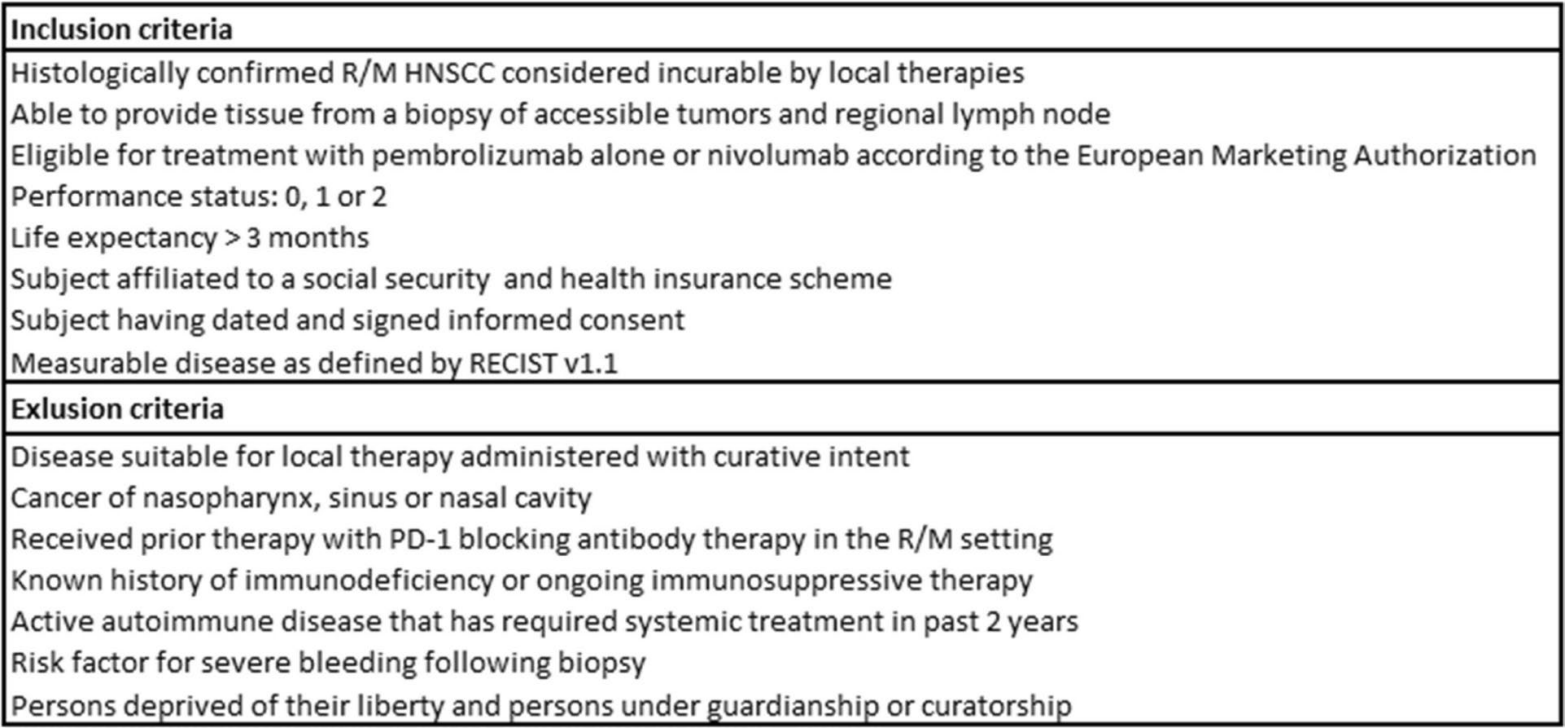
IPRICE study inclusion and exclusion criteria

### Study assessment

Clinicians will inform all the patients enrolled in the study that their biological samples could be used for research purposes and will obtain informed consent from the participants. All participating donors may object at any time, leading to the disposal of their tissues and any derived material, as well as the cessation of data collection. The enrollment period of the study will be 3 years; each patient will be monitored for 3 years and study duration will be 6 years.

### Sample processing

#### Collection of tumor samples and liquid biopsies

Prior to first-line immunotherapy, tumor biopsy will be performed as part of standard care for patients with histologically proven disease. Tumor biopsies will be performed prior to second-line immunotherapy following chemotherapy and 3 months (D84 ± 5 days) after the start of treatment. Biopsies will be proposed only if disease progression is observed and 50 days after the start of chemotherapy following immunotherapy. Samples will be processed using the standard formalin-fixed paraffin-embedded (FFPE) procedure.

Liquid biopsies will be taken before the start of anti-PD-1 immunotherapy, before and after each of the 4 cycles (20-day cycle) up to 84 days (± 4 days) after the start of immunotherapy, and finally at the end of anti-PD-1 immunotherapy (10 samples per patient). Biopsies will also be performed in the event of disease progression prior to the initiation of a new line of chemotherapy. Liquid biopsies will be collected in anti-coagulant ethylenediaminetetraacetic acid (EDTA)-treated RNA Complete blood collection tubes (BCT). Cell-free plasma samples will be isolated by a two-step centrifugation (1,200 g, 10 min and 16,000 g, 10 min) and aliquoted before storage at -80 °C in the Biobank of the UNICANCER Centre Paul Strauss in Strasbourg.

#### Tumor analysis by spatial transcriptomics and single cell RNA-seq

Spatial transcriptomics will be performed on FFPE tissue sections using the 10X Genomics Visium Spatial Gene Expression platform (10X Genomics, GenomEast, IGBMC, Illkirch, France). Histological sections will be stained using hematoxylin and eosin (H&E) to select the most relevant areas for analysis. Using a Leica RM2235 microtome (Leica, Nanterre, France), tissue sections will be cut from FFPE tissue blocks and placed within the etched frames of the capture areas on the active surface of the Visium Spatial Slide using the Visium CytAssist. The slide will be dried in an oven overnight at 37 °C and then at 60 °C for 25 min before dehydration in ethanol and H&E staining. The slide will finally be transferred to water and mounted in 87% glycerol before image acquisition on the same day. Image mosaic of each section will be acquired to generate a brightfield image of each section including the fiducial marker frame. Subsequently, stained sections will be processed as described in the demonstrated protocol “Visium Spatial Gene-Expression Reagent kits for FFPE, UserGuide” (CG000407). This kit enables spatial quantification of the expression of approximately 18,000 genes on FFPE tissue sections using a pair of specific probes for each targeted gene. Slides containing 4 capture zones will be used. Each 6.5 × 6.5 mm capture zone contains ~ 5,000 spots, and each spot is covered with capture probes bearing a unique barcode, known as a spatial barcode. Quantification and quality control of the final libraries will be performed using Bioanalyzer 2100 (Agilent Technologies, Santa Clara, United States).

Libraries will be sequenced on an Illumina NextSeq 2000 platform as paired-end 28 + 50 bases reads. Sequencing data will be processed using the Space Ranger software (10 × Genomics) for analyzing and visualizing spatial gene data produced by the 10 × Genomics Visium Platform including detection tissue, alignment reads, generation of read counts matrices, clustering and gene expression analysis, and place spots in spatial context on the microscope slide image. Differentially expressed gene (DEG) analysis between responders and non-responders will be performed using pseudo-bulking on DESeq2.

Signaling pathways and cellular/molecular processes characterized by the expression of genes identified in each spot will be identified using online bioinformatics tools (such as Database for Annotation, Visualization and Integrated Discovery (DAVID) and Search Tool for Retrieval of Interacting Genes/Proteins (STRING)).

Single cell RNA-seq analysis will also be performed on the same fixed tissues. Advances in technology have enabled RNA extraction from FFPE which had been a challenge due to RNA modification and degradation [44]. The protocol includes sample selection, paraffin dissolution, single nuclei isolation, and permeabilization, single-strand DNA blocking, reverse transcription for converting the mRNAs into cDNAs labeled with specific barcodes, and sequencing library by adding sequencing adapters and PCR amplification. A trypan blue exclusion assay will be used to determine cell number and viability using a Neubauer Chamber. Cells will be processed using the Chromium iX (10X Genomics). Chromium Single-Cell 3′ Reagent Kits (10X Genomics ref. CG00052) will be used to generate sc-3′ mRNA-seq libraries. Quality control and quantification of libraries will be performed using Bioanalyzer 2100 (Agilent Technologies). Generated libraries will be sequenced on NextSeq 2000 (Illumina, San Diego, United States) sequencer as 28 + 85 bases paired-end reads.

#### Profiling circulating miRNA from liquid biopsies

The miRNAs will be extracted from 200 μl of cell-free plasma using the miRNeasy serum/plasma extraction kit (Qiagen) according to the manufacturer’s instructions. RNA quality will be assessed using a Nanodrop Spectrophotometer (Thermo Scientific), resulting in a mean 260/280 ratio of 1.95. RNA will be processed for miRNA-sequencing and RT-qPCR. The sequencing of miRNAs extracted from the serum through liquid biopsy will be performed by Qiagen, a leading provider for RNA studies. The study will include sample quality control using qPCR, library construction and quality control, miRNA sequencing, and analysis of the results.

As a complement, digital PCR (dPCR) based on the partitioning of the sample into thousands of micro-reactions of defined volume will be performed with miRNA of interest because of higher precision and sensitivity to detect low-abundance miRNAs in plasma samples [45].

The potential predictive value of miRNAs signature will be validated. In addition, optimal expression cutoff values will be determined using receiver operating characteristic (ROC) curve analysis to determine the relationship between miRNAs signature and Overall Response Rate (ORR; used as an endpoint). Hazard ratios will be adjusted in multivariate models including the principal known confounding prognostic factors, such as patient age, tobacco smoking, treatment, co-morbidities, margin status, extra-capsular status, tumour localization, differentiation, stage, lymph node involvement, and tumor size.

#### Profiling circulating cytokine from liquid biopsies

We will measure cytokines known to be associated with inflammation, angiogenesis, and growth processes using a membrane-based Human Cytokine Antibody Array kit according to the manufacturer’s instructions.

#### Panel of immune-related genes and signatures expression analysis (non-exhaustive list)

DEG analysis between responders and non-responders to anti-PD1/PD-L1 immunotherapy will be performed including:

- IFN-γ mediated signaling
- Stimulatory (CD27, CD28, CD40, OX40, glucocorticoid-induced TNFR-related protein (GITR), inducible costimulator of T cells (ICOS),…) and Inhibitory (PD1, B7-H3, B and T lymphocyte attenuator (BTLA), cytotoxic T lymphocyte antigen 4 (CTLA-4), indoleamine 2,3-dioxygenase (IDO), killer cell immunoglobulin-like (KIR), lymphocyte-activation gene 3 (LAG3), T-cell immunoglobulin and mucin domain 3 (TIM3), V-domain Ig suppressor of T cell activation (VISTA),…) immune checkpoint genes
- Hypoxia-related genes (Hypoxia-inducible factor (HIF)-1α; HIF-2α,…)
- ΔNp63 (p40) gene expression signature
- Immune cell gene signatures
- Immunogenic cell death (Calreticulin, p-eukaryotic initiation factor 2α (eIF2α),…) and mitophagy-related markers (Bcl-2 interacting protein 3 (BNIP3),…)

### Medical data collection

The following data will be collected for each patient to correlate biological data with clinical response, at the time of inclusion and during medical follow-up: demographic and clinical data (age, sex, weight, height, performance status,…), medical history, lines of treatment, clinical evaluation, and imaging reports. All data will be recorded in the Case Report Form (CRF) (CleanWebTM, Copyright ©2023 Telemedicine Technologies).

### Statistical analysis

Primary endpoint will be the measure of response to PD-1 checkpoint blockade (overall response, CR, and PR) in patients with IFN-γ–related gene expression signature. The expected response rate in patients with IFN-γ–related gene expression signature is 60%, the expected two-sided alpha error is 0.05 and beta error is 0.2, the required number of patients is calculated to be 54 based on the exact binomial test (Casagrande et Pike) and considering the patients excluded from the analysis due to censoring. DEG analysis between responders and non-responders will be performed using DESeq2 package. IFN-γ–related gene expression signature and other genes include in the panel will be considered differentially expressed if log 2-fold change is at least ± 2 and *p*-adjusted value ≤ 0.05.

## Discussion

Immune checkpoint inhibitors of PD-(L)1 represent a significant breakthrough in the treatment of HNSCC in the R/M setting because of the prolonged responses observed with a near doubling of the 2-year survival in the Keynote-048 study. However, response rates remain < 20% with life-threatening progression, even hyperprogression occurring in as much as 30% of the cases in some studies, resulting in excess mortality observed in the initial part of the immunotherapy arms survival curves [46]. The increase of this response rate requires new immunomodulatory agents and the development of effective biomarkers to support a better selection of patients most suitable for immunotherapy. But the last few years have seen a number of failures in clinical drug development for HNSCC, which may reflect an incomplete understanding of the TME including the molecular and cellular drivers of immune escape. In this study, we consider the understanding of the dynamic evolution of TME under immune checkpoint inhibitors to be of crucial importance. This will be accomplished through the emergence of breakthrough technologies including scRNA-seq and spatial transcriptomics (performing a deep analysis with cellular resolution of the main cellular subsets), and the anatomical localization of HNSCC allowing for multiple biopsies during the treatment. However, we realize that the main limitation of this study is the TME heterogeneity. Indeed, a single tumor sample may not be representative of the immune infiltration landscape if, for example, the same tumor has both “immune cold” and “immune hot” regions which could affect up to a third of the patients [26]. Complementary approaches such as liquid biopsy could help overcome this problem, and will be included in this study to focus on the exploration of circulating miRNA-mediated immunomodulation and circulating cytokines.

In this single-center prospective study, we will study biological samples of patients undergoing anti-PD-1 immunotherapy to generate effective biomarkers to define the optimal therapeutic strategy and identify potential new immunomodulatory targets in patients with R/M HNSCC.

## Data Availability

This section is not applicable.
